# Mask R-CNN assisted 2.5D object detection pipeline of ^68^Ga-PSMA-11 PET/CT-positive metastatic pelvic lymph node after radical prostatectomy from solely CT imaging

**DOI:** 10.1038/s41598-023-28669-y

**Published:** 2023-01-30

**Authors:** Di Xu, Martin Ma, Minsong Cao, Amar U. Kishan, Nicholas G. Nickols, Fabien Scalzo, Ke Sheng

**Affiliations:** 1 Computer Science, University of California, Los Angeles, CA 90035 USA; 2 Radiation Oncology, University of California, Los Angeles, CA 90035 USA; 3 Department of Radiation Oncology, VA Greater Los Angeles Healthcare System, Los Angeles, CA 90035 USA; 4 Computer Science, Pepperdine University, 24255 Pacific Coast Hwy, Los Angeles, CA 90263 USA; 5 Department of Radiation Oncology, University of California, San Francisco, CA 94115 USA

**Keywords:** Prostate, Urology, Biomarkers, Diagnostic markers, Cancer imaging, Cancer screening, Cancer

## Abstract

Prostate-specific membrane antigen (PSMA) positron emission tomography (PET)/computed tomography (CT) is a molecular and functional imaging modality with better restaging accuracy over conventional imaging for detecting prostate cancer in men suspected of lymph node (LN) progression after definitive therapy. However, the availability of PSMA PET/CT is limited in both low-resource settings and for repeating imaging surveillance. In contrast, CT is widely available, cost-effective, and routinely performed as part of patient follow-up or radiotherapy workflow. Compared with the molecular activities, the morphological and texture changes of subclinical LNs in CT are subtle, making manual detection of positive LNs infeasible. Instead, we harness the power of artificial intelligence for automated LN detection on CT. We examined ^68^Ga-PSMA-11 PET/CT images from 88 patients (including 739 PSMA PET/CT-positive pelvic LNs) who experienced a biochemical recurrence after radical prostatectomy and presented for salvage radiotherapy with prostate-specific antigen < 1 ng/mL. Scans were divided into a training set (nPatient = 52, nNode = 400), a validation set (nPatient = 18, nNode = 143), and a test set (nPatient = 18, nNodes = 196). Using PSMA PET/CT as the ground truth and consensus pelvic LN clinical target volumes as search regions, a 2.5-dimensional (2.5D) Mask R-CNN based object detection framework was trained. The entire framework contained whole slice imaging pretraining, masked-out region fine-tuning, prediction post-processing, and “window bagging”. Following an additional preprocessing step—pelvic LN clinical target volume extraction, our pipeline located positive pelvic LNs solely based on CT scans. Our pipeline could achieve a sensitivity of 83.351%, specificity of 58.621% out of 196 positive pelvic LNs from 18 patients in the test set, of which most of the false positives can be post-removable by radiologists. Our tool may aid CT-based detection of pelvic LN metastasis and triage patients most unlikely to benefit from the PSMA PET/CT scan.

## Introduction

Prostate cancer is the second most frequently diagnosed cancer in men worldwide^1^. Radical prostatectomy (RP) is a standard of care option for all men with localized disease^2^. Unfortunately, about 20–40% of patients treated with RP will develop a biochemical recurrence (BCR) from prostate bed recurrence, pelvic lymph nodes (LNs), or distant metastases. Early detection of the disease could improve the efficacy of intervention and reduce treatment-related toxicity. The source of the prostate-specific antigen (PSA) rise includes prostate bed, pelvic LNs or distant metastases. Conventional imaging studies are thought to have low sensitivity at low PSA levels, which poses a challenge since earlier salvage radiotherapy is known to be more effective than late salvage radiotherapy^3,4^. Advanced nuclear medicine tests, such as flucicolvine^5^ and Prostate-specific membrane antigen (PSMA) positron emission tomography (PET)^6^, have a much higher sensitivity and can detect the location of recurrences at much lower PSA values. Studies have reported patient-based sensitivity and specificity of 98.7–100% and 88.2–100%, respectively^7,8^. Recently, the landmark EMPIRE-1 trial showed improved event-free survival with the incorporation of fluciclovine PET into radiation planning after RP^9^. A head-to-head trial has shown that the detection rate and sensitivity of PSMA is superior to that of Axumin for pelvic and extrapelvic disease^10^.

Unfortunately, the PSMA PET/computed tomography (CT) carries significantly higher average overall costs compared to CT scans^10^. The cost can be prohibitive in low-resource settings and/or if repeated scans are needed. Therefore, significant barriers exist for the widespread use of ^68^Ga-PSMA-11 PET/CT for detecting prostate cancer recurrence after radical prostatectomy at the present time.

Unprecedented progress has been made in artificial intelligence in the past decade, which has demonstrated great promise in many fields, including computer-aided diagnosis (CAD) of metastatic tumor spreads. Lately, researchers have been coming up with numerous solutions regarding the classification of various types of metastases^11^. For example, Zhou et al. demonstrated the feasibility of breast cancer metastases classification using convolutional neural networks (CNN)^12^, while Ariji et al. designed CNN for nodal metastases classification^13^. For metastatic prostate cancer (PCa), Hartenstein et al. presented the work of PCa LN metastasis classification^14^.

Nevertheless, most of the current CAD metastases detection methods are limited to binary patch classification with an evenly balanced mix of positive/negative cases (50%/50%), which would be difficult to apply in the clinical setting^15^. The corresponding reasons are two-fold. First, extracting incoming patients’ scanning into patches or voxels and then feeding into classification algorithms are too labor-intensive to be included into a clinical workflow. Second, artificially balanced positive to negative cases bears little resemblance to the ratio seen in the real-world setting.

Compared to most classification methodologies, modern object detection networks are more powerful tools that can identify and localize abnormalities from the entire input feature maps^16^. Lately, Zhao et al. proposed a triple-combining 2.5D U-Net pipeline for metastatic pelvis bone and LN lesion segmentation on ^68^Ga-PSMA-11 PET/CT. This framework consisted of three 2.5D U-Nets, which extracted features from axial, coronal, and sagittal planes and predicted tumor masks based on majority voting^17^. They assessed the regime with the input of CT/PET alone or a fusion of the two.

Recent object detection and localization methods could be divided into one-stage as well as two-stage approaches. One-stage models, including the YOLO series^18^ and U-Net derivatives^19^ are more efficient, whereas two-stage ones, including the R-CNN family, are of better accuracy^20^. Since most tasks in clinical practice are more rigid on the accuracy of the modality, two-stage detectors are more favorable for learning medical imaging^21^.

In the present study, built from Mask R-CNN^22^, we investigated the feasibility of detecting PCa LN metastases solely based on diagnostic CT images with contours on pelvic lymph node clinical target volumes (CTVs).

## Materials and methods

### Dataset

#### Patients and data management

In total, 88 PCa patients who showed positive lymph nodes in PSMA PET/CT at 4 institutions (the Technical University of Munich, the University of California at Los Angeles, Ludwig-Maximilians-University of Munich, and the University of Essen) were included. All patients underwent radical prostatectomy, had BCR without prior radiotherapy and underwent ^68^Ga-PSMA-11 PET/CT at a serum PSA level of less than 1 ng/mL between August 2013 and May 2017 to detect the sites of recurrence. All patients gave written consent to undergo the procedures. The clinical data and Digital Imaging and Communications in Medicine (DICOM) files of all patients were anonymized and imported onto a dedicated radiotherapy contouring workstation at UCLA (MIM, version 6.7.5; MIM Software Inc., location of the company). This post hoc retrospective analysis was approved by the UCLA Institutional Review Board (#12-001882), and the requirement to obtain informed consent was waived. All experiments were performed in accordance with relevant guidelines and regulations.

#### ^68^Ga-PSMA-11 PET/CT image acquisition

^68^Ga-PSMA-11 PET/CT imaging was performed according to recent guidelines^23^. Images were acquired on the Siemens Biograph 128 mCT (68%), Siemens Biograph 64 (19%), Siemens Biograph 64 mCT (9%), and GE Healthcare Discovery 690 (5%). The ^68^Ga-PSMA tracer was used at all sites. The median injected dose was 154 MBq (range 65–267 MBq). To reduce bladder activity, patients received 20 mg of furosemide at the time of tracer injection if there was no contraindication^24^. The median uptake period was 59 min (range 37–132 min). A diagnostic CT scan (200–240 mAs, 120 kV) was performed after intravenous injection of contrast agent, followed by whole-body PET image acquisition (2–4 min/bed position)^25^.

#### Pelvic lymph node clinical target volumes and PET lesion contouring

Pelvic lymph node CTVs were contoured on the CT dataset of the PET/CT scan for all 88 patients by an experienced radiation oncologist who was masked to the PET findings in accordance with the radiation therapy oncology group (RTOG) consensus contouring^26,27^. CTV is a term commonly used in radiotherapy. CTV includes all at-risk LNs plus a margin for micro diseases in this specific context. We also noticed in certain cases the pelvic LNs were located at the boundary of pelvic nodal CTVs following RTOG guidelines (slightly fall out of the RTOG contours for 1–2 pixels). To ensure that the pelvic LN masks cover all the pixels of LN metastases and, more importantly, overcome the weak learning capability of CNN filter on edges of a feature map, we isotropically expanded pelvic LN CTVs by 10 absolute pixels (l = 6.48 mm). These wider contours introduced false positives (FPs) within the expansion zone but then eliminated them at the stage of post-processing (see details in “Modeling pipeline”). ^68^Ga-PSMA-11 positive lesions were contoured on the CT images by radiation oncologists. These contours were subsequently used to define ^68^Ga-PSMA-11-based target volumes^25^.

#### Data split

The patients were divided into training (nPatient = 52, nNode = 400, split ratio = 3/5), validation (nPatient = 18, nNode = 143, split ratio = 1/5), and test (nPatient = 18, nNodes = 196, split ratio = 1/5) sets balanced on their national comprehensive cancer network (NCCN) risk groups at initial diagnosis. Details of split on NCCN risk group see in Table 1.

**Table 1:** Patient split of training, validation, and test sets on NCCN risk groups.

Risk group	Training	Validation	Test
Metastatic	31	8	9
High	13	5	6
Intermidate	7	4	2
Unknown	1	1	1
In total	52	18	18

#### Windowing analysis

To narrow down the area of metastatic LN detection and accentuate the morphological features of metastases, we focused on the area inside the pelvic CTVs and carefully selected windowing strategies of Hounsfield units (HUs) during training. Table 2 lists the representative statistics of window width. Noteworthy, various ranges of widow width were selected by first conducting distribution analysis of all HUs of positive node pixels in the training set and then gradually and symmetrically excluding some extreme image pixel values at the left and right tails of the distribution based on quantile analysis. We will explore different PCa LNs metastases Hounsfield unit (HU) window width along with standard soft tissue HU widow width (− 125, 225) in the below modeling pipeline. This windowing logic will be referred to as quantile windowing strategies in the following sections.

**Table 2:** Descriptive statistics of HU distribution with different quantile ranges for PCa LNs masks and metastases.

Quantile range (%)	PCa LNs metastases
0.5–99.5	− 97 to 310
1–99	− 90 to 169
1.5–98.5	− 84 to 151
2–98	− 79 to 141

### 2.5-Dimensional (2.5D) object detection pipeline

#### Data preprocessing

**Figure 1:**
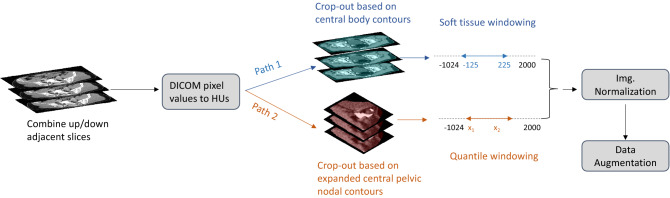
The workflow for data preprocessing. The gray box-bounded steps are identical procedures shared by WSI- as well as Regional-Mask R-CNN. The blue- and brown-marked paths are solely used for WSI- or Regional-Mask R-CNN, respectively. We compressed the shared steps for visualization convenience only.

As shown in Fig. 1, Our data preprocessing pipeline consists of two paths for images fed into the pretrained network and the fine-tuned model, respectively. For the path of pretrained processing, we performed 2.5D concatenation, HU transformation, black border crop-out, and soft tissue windowing sequentially. For that of fine-tuned processing, we performed 2.5D concatenation, HU transformation, LN CTV contour mask- and crop-out, and quantile windowing strategies. Specifically, 2.5D here means that we will channel-wise concatenate the central CT slice along with its adjacent superior/inferior slices. HU transformation is to convert the DICOM pixels stored in the bundled “three-channel” images into HUs, and LN CTV contour mask- and crop-out operation set the pixels outside of the expanded central pelvic nodal contours on CT to zero and crop the image to only keep the CTV region so as to ease the fine-tune learning process.

After the above procedures, we wrap up both paths by performing uniform normalization and data augmentation of the images. The data is geometrically augmented using random resizing (image largest width to 640–800), horizontal flipping (*p* = 0.5) and random rotation (angle 0–180°), and morphologically augmented using random gaussian noise (kernel = 5, sigma = 1) and random brightness.

#### Modeling pipeline

**Figure 2:**
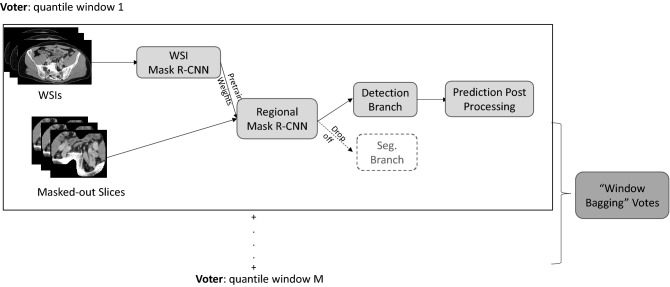
The pipeline for our proposed 2.5D object recognition mechanism of metastatic PCa LNs. Windowing strategies 1*…M* represents different HU windowing strategies.

As shown in Fig. 2, the complete design of workflow includes three steps, the initial pretrained whole slice imaging (WSI)-Mask R-CNN, the further fine-tuned Regional Mask R-CNN, and the “window bagging”. Our rationales will be elaborated on in below.

##### Pretraining to fine tuning

For Mask R-CNN with ResNet-X^28^ backbones*,* researchers commonly use weights pretrained on ImageNet^29^. Nevertheless, limited by our small set of training data, we suspect that directly training from the ImageNet pretrained weights might not lead to model convergence but instead overfit the current training set. Therefore, we designed a pretraining-to-finetuning workflow to maximize the information our model could extract from the limited training set. In the pretraining stage, we input the detection network with WSIs—CT scans without LNs CTV mask-out—and performed a quick and dirty training to let the model grasp initial coarse morphological structures in the patients’ pelvic CTs dataset (WSI-Mask R-CNN). Next, in the fine-tuning stage, by training the detector with the input of the pelvic nodal masked-out slices loaded with WSI pretrained weights (Regional-Mask R-CNN), we improved the starting point for a better chance of reaching the global optima with back-propagation. Since we will only perform object detection in this specific task, we blocked off the mask-branch of the standard Mask-R-CNN network in both the pretraining and fine-tuning stages. Modeling details can be seen in Fig. 2.

##### Prediction post-processing

During experiments, we found that our Regional-Mask R-CNN still suffered from two types of false positives—predictions near the outer boundary of expansion zone and vascular/bowel structures—that could benefit from post-processing. Three hyper-parameters (see $\tau$_4−6_ in Table 3) were cross-validated to automate the post-processing. For FPs of the expansion zone boundary, we set ${\tau }_{4}$ to regularize the valid predictable LNs nodal expansion zone from a range of 1–10 pixels. For vascular/bowel structure FPs, we set $\tau$_5−6_ to determine the quantile of all HUs within the predicted detection box (${\tau }_{6}$) above which threshold of HUs (${\tau }_{5}$) was not taken in the final prediction set. Since vessel and bowel patterns both have higher HUs than pelvic nodes on contract enhanced CTs.

**Table 3:** Tunable hyper-parameters elaboration for the paragraphs of prediction post-processing and “bagging” in Sect. 2.2.2.

Hyper-parameters	Explanation	Tuning range	Section
τ1	IoU threshold for prediction filtering	[0.1, 0.2, 0.3, …, 1.0]	Window bagging
τ2	Threshold for including final voter numbers	[1–5]	Window bagging
τ3	IoU threshold for determination of bagged prediction hitting GTs	[0.1, 0.2, 0.3, …, 1.0]	Window bagging
τ4	Threshold for filtering predicted detection box in expansion zone	[1, 2, 3, …, 10]	Post-processing
τ5	Absolute threshold for filtering HU	[80, 100, …, 160]	Post-processing
τ6	Quantile threshold for filtering HU	[0.1, 0.2, 0.3, …, 1.0]	Post-processing

#####  “Window bagging”

To further enhance model performance, we bagged multiple post-processed Regional Mask R-CNN trained with different quantile windowing inputs, the so-called “window bagging”, to count the votes from the crowd. Notably, bootstrap of the dataset was not conducted here for each voter since we believe that inputs with different quantile windowing could diversify the training information and therefore avoid collinearity. Details of our “window bagging” workflow can be seen in Fig. 2.

${\tau }_{1-3}$ are cross-validated hyper-parameters for “window bagging” tuning. ${\tau }_{1}$ is the intersection over union (IoU) threshold for determining the detection boxes generated from different voters as the final “window bagging” prediction. ${\tau }_{2}$ decides the number of voters in the final “window bagging” models. ${\tau }_{3}$ is the IoU for recognition of whether the bagged prediction hits ground truths (GTs).

#### Loss function

Although hybrid loss functions have been used recently in various deep networks^30–32^, our loss function kept the same as the original Mask R-CNN due to its efficiency with the dataset.

1 $$L={L}_{cls}+{L}_{box}+{L}_{mask}$$

where ${L}_{cls}$ and ${L}_{box}$ still follows the definition in Faster R-CNN^33^ and ${L}_{mask}$ is the average binary cross entropy loss proposed in Mask R-CNN^22^.

### Model training

**Figure 3:**
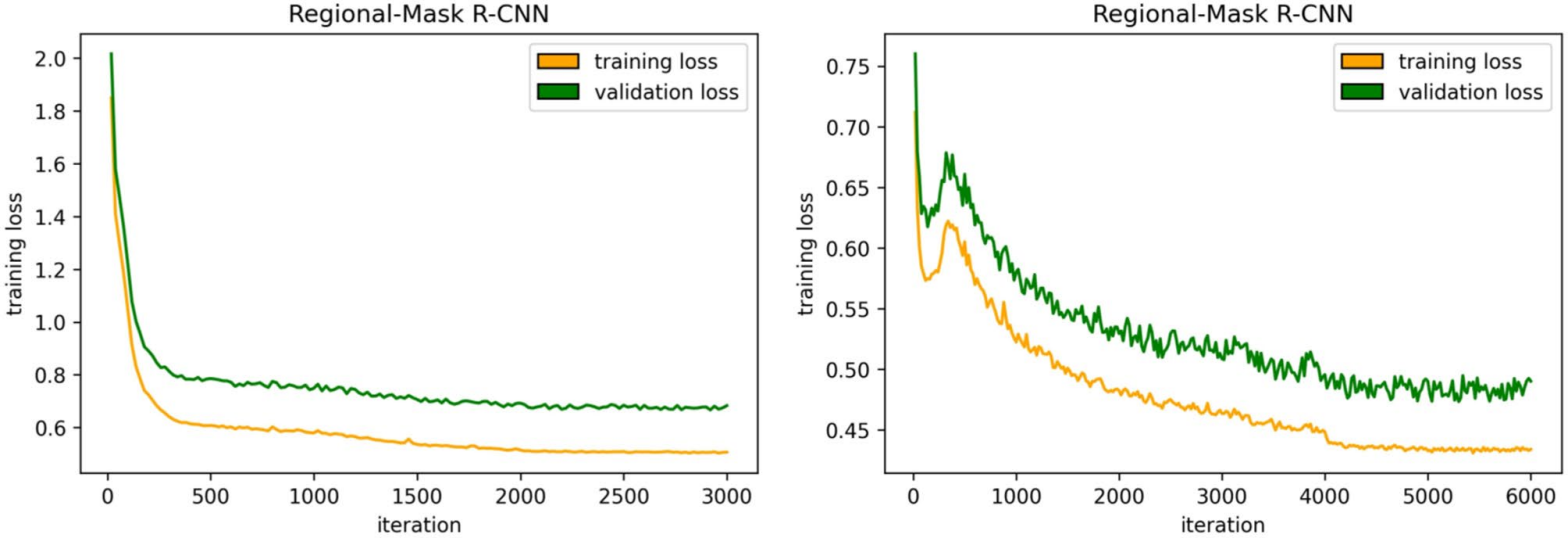
Training and validation losses convergence plots, the left-hand-side plot is an example of the WSI Mask R-CNN model training, and the right-hand-side one is an instance of the training of the Regional Mask R-CNN model.

Our 2.5D object detection pipeline was implemented in detectron2 (https://github.com/facebookresearch/detectron2) project using PyTorch and performed on a GPU cluster with 4 × RTXA6000. Figure 3 shows the two training processes in detail.

For WSI Mask R-CNN, we trained the three-channel whole slice images on stochastic gradient descent (SGD) optimizer for 3 k iterations, with a batch size of 64 (4 $\times$ 16), learning rate (LR) of 0.01 decreasing by tenfold at 2 k iterations, a momentum of 0.9, and weight decay of 0.0001.

For Regional Mask R-CNN, we fine-tuned the pelvic nodal contour masked-out three-channel images using SGD for 6 k iterations with a batch size of 64 (4 $\times$ 16), LR of 0.005 decreasing by tenfold at 4 k and 5 k iterations, respectively, a momentum of 0.9, and weight decay of 0.0001. The final training loss decreased to around 0.4.

### Model evaluation

We reported the best performance, tuned from individual criteria, including sensitivity, precision, and F-1 score for steps of prediction post-processing and “window-bagging”. Sensitivity is defined at the metastasis level, which means that if the model could locate one slice of a single metastatic LNs, we count this entire metastasis as a hit. Precision is defined as the slice level, which counts each slice of metastases captured by the detection box predictions. All metrics are evaluated on node instead of patient level.

## Results

**Figure 4:**
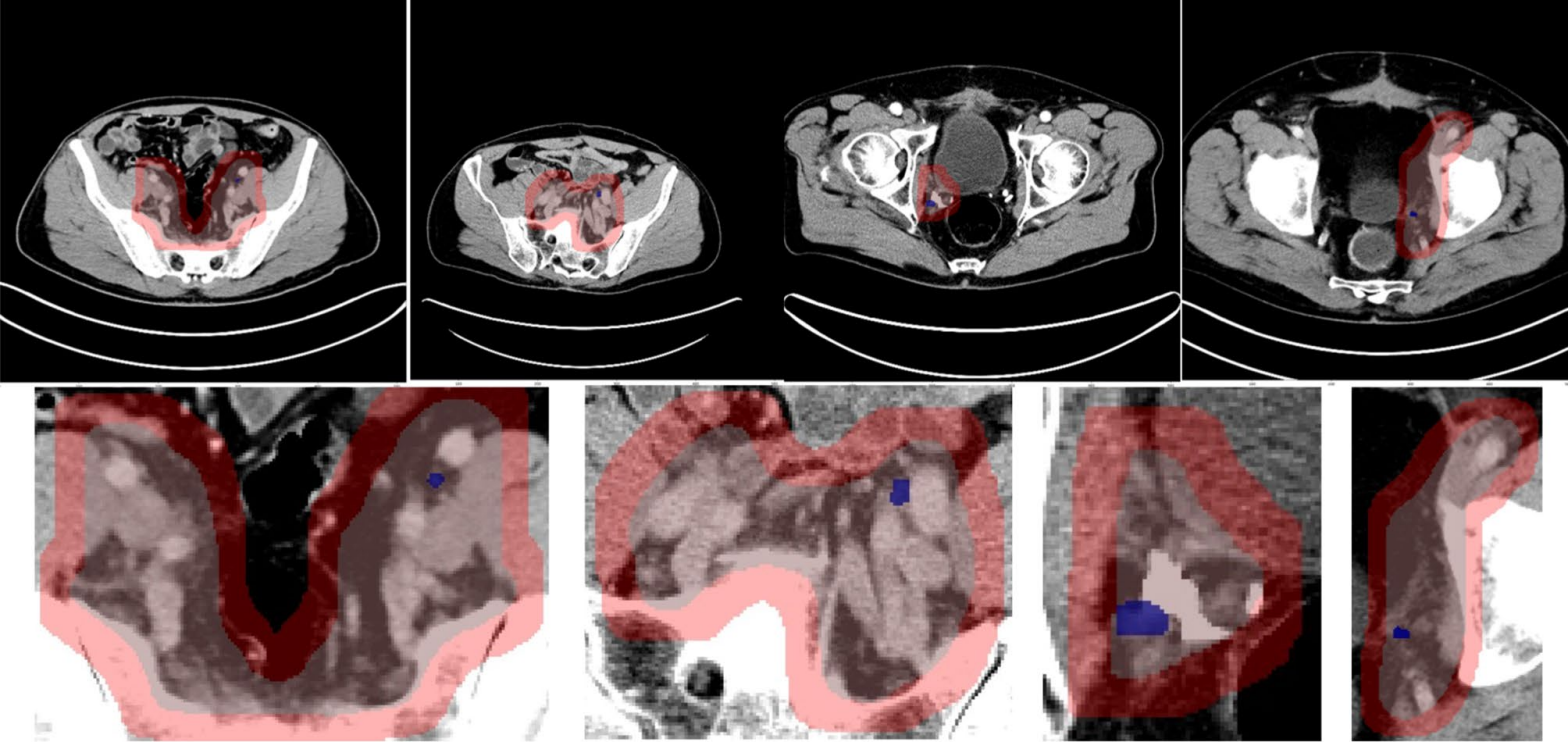
Visualization of positive pelvic LN GT and CTV masks. Below are two-image-paired examples selected from four different patients. For each pair, the first row is WSI with light red mask showing RTOG pelvic LN CTV and dark red mask showing the expanded zones, the second row is the enlarged subregion of its corresponding patient to better visualize the positive metastatic tumors (in blue).

**Figure 5:**
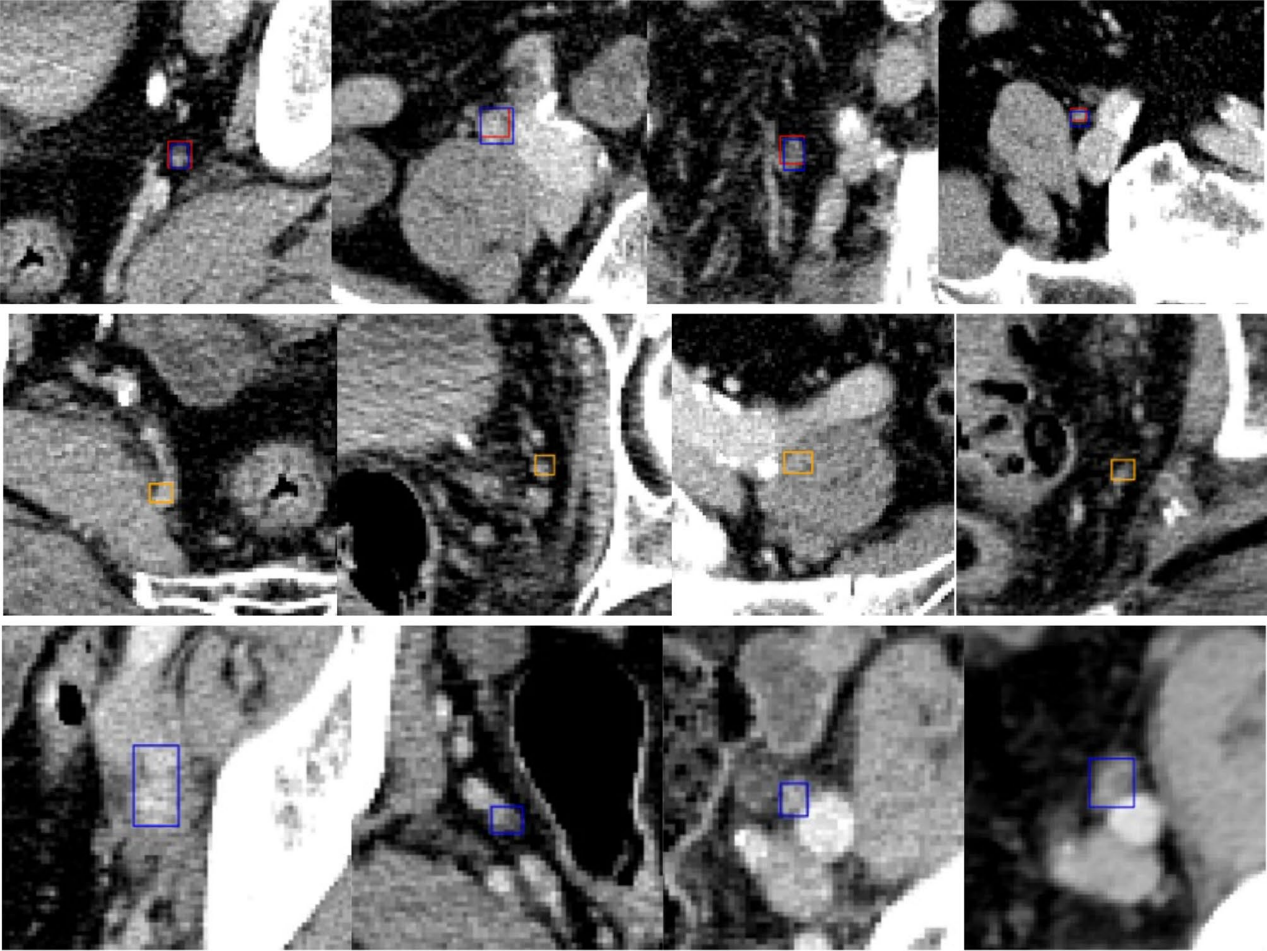
Examples of 2.5D Object Detection Pipeline Prediction on the test set. Images are zoomed in to better visualize the detection boxes. Boxes in red are TPs, in orange are FPs, in blue are GTs. The first row is the visualization of TPs, the second and last are for FPs and FNs, respectively.

Positive pelvic LN GTs with the CTV contours are visualized in Fig. 4. Qualitative and quantitative results are presented in Fig. 5 and Table 4, respectively. Figure 5 enlarges the representative 2D images to highlight the sub-regions near the predictive or ground-truth positive LNs, and the detection boxes. Note that a positive LN can be found in multiple adjacent 2D slices, and a number of positive LNs could apprear in one slice. Visually from Fig. 5, there is not a clear difference between true positives (TPs), FPs, and false negatives (FNs), showing the challenge of directly using the CT for manual lymph node detection and classification.

**Table 4:** Performance comparison and ablation studies on the test set of ^68^Ga-PSMA-11 PET/CT.

WSI pretrain	Post-processing	Bagging	Metrics	(− 79,141) (%)	Quantile (− 84, 151) (%)	Windowing (− 90, 169) (%)	(− 125, 225) (%)	(− 97, 310) (%)
			Sensitivity	66.671	75.831	70.831	75.002	70.831
			Precision	26.743	28.642	28.844	25.896	24.000
			F-1 Score	38.17	41.453	41.323	38.498	35.855
			AUC	79.667	87.231	84.327	87.092	84.215
			Sensitivity	66.674	70.834	70.836	83.333	75.006
✓	✓		Precision	31.716	29.825	32.068	29.573	30.101
			F-1 Score	42.987	41.976	44.146	43.651	42.968
			AUC	80.765	84.799	83.972	89.124	86.998
			Best sensitivity	62.501	66.671	66.678	83.333	66.679
✓	✓		Best precision	47.942	47.943	47.957	48.945	47.954
			Best F-1 Score	48.854	52.382	52.382	53.077	52.045
			AUC	78.257	80.011	79.993	89.579	79.681
			Best sensitivity	**83.351**				
✓	✓	✓	Best precision	**58.621**				
			Best F-1 Score	**60.021**				
			AUC	**90.034**				

Bold results are the best ones. Underlined results are the worst cases, indicating the effectiveness of ablated strategies. Sensitivity, Precision, F-1 score, and area under the curve (AUC) are used as evaluation metrics. Specifically, AUC is calculated image-based, where the probabilities of any detected nodes for an image will be examined, and the maximum of those nodules will be chosen as an image score, and if there is no prediction for a certain image, we set the corresponding image score as 0. Results in the last three rows are generated from “window bagging” of the model predictions of all quantile windowing ranges listed in the below columns.

Table 4 shows a quantitative comparison of detection methods. The single ImageNet-pretrained Regional Mask R-CNN resulted in robust sensitivity achieving ~ 80% AUC and detecting > 60% of the positive LNs but low precision under 30%. Fine-tuning individual Regional Mask R-CNNs from weights of WSI Mask R-CNN improves the precision by ~ 5% without compromising sensitivity. Prediction post-processing improved each learner by another 15%. Lastly, via “window bagging” of Regional Mask R-CNN pretrained on WSI as well as prediction post-processing, we obtained another 5% gain in precision score with a high sensitivity of 83.351% and AUC of 90.034%.

## Discussion and conclusion

In the study, we developed a 2.5D deep learning pipeline for prostate metastatic LNs. As shown in Fig. 5, the differences between negative and positive nodes are subtle in CT, making it impractical for human observers to perform the detection task. However, after supervised learning based on PSMA-PET, our AI pipeline located the majority of positive pelvic LNs solely based on pelvic LN region extracted from CT scans, achieving an AUC of 90.034%, sensitivity of 83.351% and specificity of 58.621% out of 196 positive pelvic LNs (18 patients) in the test set. Our results show more promising performance compared to the triple-combining 2.5D U-Net proposed by Zhao et al., where the specificity of 54.8% and positive predictive value of 59.7% were reported for the case where solely CT was input to their network^17^.

Object detection of metastatic PCa lymph nodes using WSI CT scans is a challenging task mainly due to the enormous class imbalance between positive and negative voxels, the almost identical morphological patterns between abnormal and normal LNs, the large variance of appearances of the normal and abnormal tissues, the interference from complex pelvic structures (vascular, bowel, and pelvic bone structures), the infeasibility to balance positive and negative LNs on a WSI, and, in this specific task, a relatively small dataset to train the deep learning network. Nevertheless, our object detection pipeline still achieved superior sensitivity and relatively lower specificity than the easier binary classification problem.

We combatted those facts with five strategies: transfer learning from WSI imaging, fine tuning from regional pelvic LN CTVs, prediction post-processing, and “window bagging”. Our results show an additive and progressive improvement indicating independent mechanisms with these strategies (1) pretraining on entire CT slices provides more background information; (2) precise regional searching within CTVs greatly simplifies the complexity of feature learning; (3) prediction post-processing with tuned hyper-parameters helps refine the spatial and pixel-wise search regions; (4) “window bagging” of voters synthesizes individual training cohorts to reduce FPs while improving the robustness of sensitivity.

The present study has important clinical implications. Pelvic LN recurrence after definitive local therapy can be treated with external beam radiation therapy with or without androgen deprivation therapy. Many studies have demonstrated good efficacy and safety profile of whole pelvic radiation with simultaneous integrated boost to lymph nodes with gross disease^3,34^. Another more targeted yet experimental approach is to deliver stereotactic body radiation therapy specifically to individual lymph nodes that are involved without irradiating the pelvic lymph node region comprehensively^35–37^. In either approach, detailed information regarding the location of pelvic LNs harboring PCa is essential for treatment planning. Traditional CT-based detection method largely relies on morphological characteristics of the LNs, such as size (≥ 9–10 mm), presence of fatty hilum, shape (oval vs. round), and the short/long axis ratio^38^. PSMA PET/CT was able to detect LN metastasis in nodes under 10 mm in size, with one study reporting a 60% detection rate for nodes between 2 and 5 mm^39^. Patients with lower Gleason score (GS) tended to have smaller PSMA–positive LNs (mean 7.7 mm), than patients with intermediate- (mean 9.4 mm) and high GS cohorts. Based on the CT morphology criteria, only 34% of low GS patients, 56% of intermediate GS patients, and 53% of high GS patients were considered CT positive^40^. The examples shown in Fig. 5 confirm the challenge of visually detecting positive lymph nodes.

As PSMA PET/CT has yet to become widely available due to financial and availability barriers, a low cost and easily accessible alternative approach that can help predict the presence and location of potential pelvic LN involvement based solely on conventional diagnostic CT is extremely appealing. The method developed here is not intended to replace PSMA PET/CT. Rather, it may help clinicians select patients who may benefit the most from PSMA PET/CT. The high accuracy of classifying patients with or without positive LNs is conducive for such a task.

The current dataset with 52 training patients is still far from sufficient, leaving space to further reduce the FPs and FNs with more training data. Additionally, the current pipeline benefits from manual pelvic LNs CTV segmentation that helps focus on a smaller and more relevant search volume. However, manual labeling of the structure can be inconsistent. Moreover, LN CTVs for radiotherapy purposes do not precisely delineate the individual pelvis lymph nodes. Additional non-LN tissues are included in the CTV, complicating the detection task. In the future, an automated pelvis LN segmentation network can be trained to improve both aspects based on curated CT with detailed labeling of the structure, such as the data released by the CAMELYON17 challenges. We also plan to apply more complex z-dimensional slice fusion strategies to provide more context information for the network and adding more background information via pretraining from other datasets, including DeepLesion^41^, Luna16^42^ and etc. In addition, adding attention gating into the network is another direction to explore. Lastly, as an extension of this work, the performance of our proposed approach can be compared with the performance of a capsule network since capsule networks can preserve spatial relationships of learned features and have been proposed recently for image classification tasks^43–45^.

Another limitation of the study is that the PSMA PET is not a perfect ground truth for training and validation. PSMA PET detection sensitivity has been reported between 40 and 60% in a study^46^ for patients with low PSA levels. However, the same method used in the study should be applicable as enhanced diagnostic information from histopathology and complementary imaging modalities, e.g., hyperpolarized C-13 MRI, becomes available.

## Data Availability

The datasets generated and/or analysed during the current study are not publicly available due to a confidentiality agreement associated with using these data and institutional policy but are available from the corresponding author on reasonable request with a legal data transfer agreement between institutions.

## Code availability

The code for implementing this project is open-sourced at https://github.com/FluteXu/PSMA-Detection.
